# PSYChosomatic Medicine in ONcologIc and Cardiac Disease (PSYCHONIC) Study—A Retrospective and Prospective Observational Research Protocol

**DOI:** 10.3390/jcm10245786

**Published:** 2021-12-10

**Authors:** Adriana Roncella, Christian Pristipino, Oretta Di Carlo, Matteo Ansuini, Angela Corbosiero, Stefania Angela Di Fusco, Gabriella Palumbo, Antonella Gigantesco, Fiorino Mirabella, Rosanna De Angelis, Vincenzo Pasceri, Laura Cancellara, Furio Colivicchi, Robert Allan, Maria Alessandra Mirri, Giulio Speciale

**Affiliations:** 1 Intensive and Interventional Cardiology Unit, San Filippo Neri Hospital, 00100 Rome, Italy; adrianaroncella@hotmail.it (A.R.); matteoansuini@gmail.com (M.A.); vpasceri@hotmail.com (V.P.); lauracancellara@gmail.com (L.C.); giuliospeciale@yahoo.it (G.S.); 2 International Association of Ontopsychology (AIO), 00100 Rome, Italy; gabriella.palumbo@iss.it (G.P.); rosanna1.deangelis@gmail.com (R.D.A.); 3 Department of Radiation Oncology, San Filippo Neri Hospital, 00100 Rome, Italy; info@formaeazione.it (O.D.C.); angela.c@virgilio.it (A.C.); mamirri@inwind.it (M.A.M.); 4 Cardiology Unit, San Filippo Neri Hospital, 00100 Rome, Italy; doctstefania@hotmail.com (S.A.D.F.); furio.colivicchi@aslroma1.it (F.C.); 5 Italian National Institute of Health Center for Behavioural Sciences and Mental Health, 00100 Rome, Italy; antonella.gigantesco@iss.it (A.G.); fiorino.mirabella@iss.it (F.M.); 6 Division of Cardiology, New York-Presbyterian Hospital, Weill Cornell Medical College, New York, NY 10065, USA; cardiacpsych@msn.com

**Keywords:** ontopsychology, psychotherapy, dreams, acute myocardial infarction, takotsubo syndrome, breast cancer

## Abstract

Psychosocial factors play an important role in non-communicable diseases (NCDs). This observational study is primarily aimed at assessing the relationship of psychological characteristics of patients with the outcomes of different NCDs, and to assess short-term psychotherapy (STP) efficacy in the real world. **Methods**: One hundred and forty patients with recent acute myocardial infarction, Takotsubo syndrome, or non-metastatic breast cancer and a control group of 140 age and sex-matched healthy subjects, will be enrolled. All subjects will be administered psychometric tests, quality of life tests, a specific body perception questionnaire, a dream questionnaire, and a projective test, the Six Drawing test at baseline and follow-up. All subjects with medical conditions will be asked to freely choose between an ontopsychological STP along with standard medical therapy and, whenever indicated, rehabilitation therapy or medical therapy plus rehabilitation alone. The study endpoints will be to evaluate: the relationship of the psychological characteristics of enrolled subjects with the outcomes of different NCDs, predictors of the choice of psychotherapy, and the efficacy of ontopsychological intervention on psychological and medical outcomes. **Conclusion**: This study will generate data on distinctive psychological characteristics of patients suffering from different CDs and their relationship with medical outcomes, as well as explore the efficacy of ontopsychological STP in these patients in the real world. (Number of registration: NCT03437642).

## 1. Introduction

Non-Communicable Diseases (NCDs) represent 71% of all global deaths, the two most prevalent being cardiovascular diseases (CVD) and cancer [1]. As such, psychosocial factors have gained a rising interest as therapeutic targets in NCDs.

Indeed, several psychological conditions can predispose, precipitate, and increase the risk of some of the most ominous cardiovascular diseases, such as ischemic heart disease (IHD) [2,3].

In oncologic disease, although there are no conclusive data providing a causal link with psychological variables, psychological factors play a pivotal role in diagnosis and therapy [4]. Therefore, systematic screening for distress and supportive care needs may be recommended for these patients.

Several studies have assessed psychotherapy intervention in cardiac patients in order to reduce stress and depression and improve medical prognosis [5,6,7,8], including morbidity and mortality, reported in a recent meta-analysis of randomized controlled trials for cardiac rehabilitation (CR) programs [9]. Recently, we demonstrated in the STEP-IN-AMI (Short TErm Psychotherapy IN Acute Myocardial Infarction) trial that adding short-term ontopsychological psychotherapy (STP) to standard medical therapy in post-acute myocardial infarction (AMI) patients improved psychological symptoms and quality of life at one year [10], as well as a composite endpoint of medical and cardiac outcomes at five years [11] independently of CR. However, studies are also needed to assess the generalizability of ontopsychological psychotherapy in IHD and control for possible bias due to randomization in psychological interventions.

Moreover, sub-analysis of STEP-IN-AMI trial data provided original insights on the relationship between emotions, dreams, and medical conditions suggesting new potential diagnostic, preventive, and therapeutic strategies. As this possibility may potentially extend to other NCDs, two clinical diagnoses appear to be ideal initial candidates to test this hypothesis in addition to ischemic patients: Takotsubo syndrome and breast cancer.

Takotsubo syndrome (TTS) often mimics acute myocardial infarction, but it is not caused by an obstruction of epicardial coronary arteries and generally spontaneously heals in survivors [12,13,14,15,16]. In many cases, there is a clear and identifiable psychological or physical “trigger” for TTS. Surprisingly, no psychotherapeutic approach has been attempted in this patient population.

Breast cancer (BC) is also an ideal case study because it has been extensively investigated from a psychosocial point of view and represents a public health priority in women worldwide. Several psychological factors such as depression, hopelessness [17], anxiety, intrusive thoughts, social support [18,19], fear of recurrence [20], compassion [21], and sentimental issues [22] are known to play a complex role in the follow-up of patients with breast cancer, interfering with the quality of life, therapeutic choices, and worsening prognosis. Patients’ experience of therapeutic side effects can deeply affect body image and self-esteem [23,24,25], and while reconstructive surgery is of great importance [26], it is not known whether such surgery is sufficient to generate an experience of comprehensive healing which may impact treatment outcome.

Based on previous data from the STEP-IN-AMI trial, we have designed an observational study to obtain additional psychosomatic insights and assess the effects of ontopsychological STP in acute MI, Takotsubo, and breast cancer patients.

## 2. Materials and Methods

PSYCHONIC (PSYChosomatic medicine in ONcologIc and Cardiac disease), is a retrospective and prospective observational case-control study, approved by the relevant ethical committee (Comitato Etico Lazio 1) on 12 May 2017, amended on 9 October 2017, and registered in www.clinicaltrial.gov on 27 March 2018 (number of registration: NCT03437642). The study is compliant with the Code of Helsinki.

### 2.1. Aims

(1) To prospectively assess the clinical and psychological characteristics at enrollment, and the real-world outcomes at one and five years in patients (pts) with AMI, TTS, and BC who (a) have undergone ontopsychological STP or (b) declined the treatment, as well as (c) a control group of age/sex-matched subjects without major active diseases (for a practical exposition they are referred to as “Healthy Subjects”, HS) not undergoing psychotherapy at enrollment
(2) To assess:
  - The perception of their body and emotions in each group at baseline and follow-up;
  - Dream symbols and patterns in each group, including retrospective evaluation of past dreams (until childhood and adolescence), at baseline and follow-up;
  - Specific psychodynamic patterns in each group based on personal history, dream analysis, psychometric tests, and the projective non-structured Six Drawing test (6DT) [27,28];
  - The correlation of past dream symbols and patterns with the onset of disease in each group;
  - The correlation between baseline psychological data/evaluations and study outcomes.

#### The Rationale for the Study Aims

As specified above, the PSYCHONIC study has been inspired by the results of the previously published STEP-IN-AMI trial (Short TErm Psychotherapy IN Acute Myocardial Infarction).

The STEP-IN-AMI trial randomized 101 pts with AMI to standard medical therapy or standard care plus original short-term psychotherapy derived from the ontopsychological method (STP) [10]. Ontopsychological psychotherapy is a humanistic existential-psychotherapy associated with psychodynamic analysis [29] (for a detailed description see the paragraph dedicated to “therapies”). Compared to controls, patients who underwent STP showed a statistically significant improvement in psychological symptoms (reduction in depression scores, assessed by the Beck Depression Inventory {BDI}) [30,31], improvement in quality of life at a one-year follow-up as assessed by the MacNew Heart Disease Health-Related Quality of Life Questionnaire [32], and a statistically significant improvement in medical and cardiac prognosis (reduction of angina incidence, new comorbidities, and new admissions for cardiac and other medical events) at five years’ time [11]. Despite the high internal validity of this study, ontopsychological STP outcomes remain to be assessed in the real world where the active choice of therapy by the patient has a pivotal role in intervention efficacy.

Moreover, the encouraging results of the STEP-IN-AMI trial suggest that several specific psychosomatic issues deserve further evaluation in a wider clinical context to assess new potential diagnostic, preventive, and therapeutic strategies which may potentially extend to other diseases. Particularly, the study analysis revealed for the first time two main psychological patterns:

(1) Poor awareness of emotions, which may have reflected a lack of the perception of one’s body, or parts of one’s body. Patients seemed to have a painful perception of parts of their body (mainly the head and/or the chest, with particular reference to their heart), whereas they did not have a perception of other parts, as if they were “anesthetized”. In the STEP-IN-AMI pts, there was an improvement of body/emotional perception during psychotherapeutic intervention with body relaxation training [33], conducted both in individual and group meetings. Patients were encouraged to explore their bodily sensations, starting with the visceral zone (i.e., abdomen), with the help of abdominal breathing. From our experience, abdominal breathing seems to act as a strong stimulus to the visceral brain, which is continuously active throughout one’s entire life (even if we have lost our conscious perception of it), and the subjective contact with the visceral zone translates as an important mediator of inner emotions and feelings [34].
(2) An apparent presence of specific dream configurations in the period before AMI (i.e., distressing dreams or repeated nightmares; no memory of dream content; or selective memory only of dreams of childhood and adolescence), which again improved when recovering from the acute event in those undergoing STP [10,11].

Following observations from ontopsychology, the dream pattern seems to follow a hierarchical approach [27]. In particular, the unconscious dynamics of the dream seem to primarily aim at the optimal psycho-physical evolution of the dreamer, the basic one being the biological aspect represented by the dreamer’s health. Following this point of view, at least in our observations, specific dream patterns may be ahead by months or years of the acute cardiac event as if the dream symbols reflect how the dreamer is behaving towards his/her health (concerning both lifestyle and choices/decisions in his/her existential context).

No studies have addressed the issue related to dream patterns in cardiac patients, and in psychosomatics in general.

Similarly, no studies have addressed a systematic and comprehensive psychodynamic profile of patients suffering from different diseases to guide prevention and therapies.

### 2.2. Participants

#### 2.2.1. Inclusion Criteria

(1) Patients with AMI with ST-elevation on the admission electrocardiogram (STEMI) and without ST elevation (NSTEMI) treated with urgent percutaneous coronary intervention (PCI); up to 12 h for STEMI and 48 h for NSTEMI patients.

According to the Fourth Universal Definition of Myocardial Infarction, acute myocardial injury is defined as the presence of either positive troponins with a rise-and-fall pattern, a fall-only pattern (in case the first available troponin was the peak), or a rise-only pattern [35,36].

(2) Patients with TTS

Diagnosis of TTS is based on the modified Mayo Clinic Diagnostic Criteria including (1) transient wall motion abnormality of the left ventricle with extension beyond a single epicardial coronary artery distribution; (2) absence of obstructive coronary artery disease or angiographic evidence of acute plaque rupture; (3) new electrocardiographic abnormalities or troponin value increase; and 4) the absence of myocarditis or pheochromocytoma at index hospitalization [12,37,38].

Although TTS was initially described with angiographically normal coronary arteries, smaller studies recently indicated a potential coexistence of coronary artery disease (CAD) in TTS patients [39]. To avoid confusion in the initial diagnosis, in this protocol TTS pts without critical coronary artery stenosis will be enrolled.

(3) Patients recently operated on for non-metastatic BC treated with radiotherapy and/or chemotherapy and/or hormone therapy.
(4) A control group of subjects without major active diseases (HS), matched for age and sex. This includes subjects without clinical evidence of major diseases that required hospitalization in the last ten years or chronic medical therapy (e.g., anti-neoplastic, immunosuppressant, cortisone, psychiatric, etc.), and not undergoing or scheduled for psychotherapy at enrollment.

Inclusion criteria for all patient groups include an age between 30 and 75 years old, the ability to provide written informed consent to participate in the study, and availability to undergo psychotherapeutic treatment.

#### 2.2.2. Exclusion Criteria

Patients with a disability, cognitive impairment, life-threatening conditions, or enrollment in another clinical study will be excluded.

##### Sample Sizing

The size of the AMI group has been based on evaluation of the outcome data at one year from the results of the STEP-IN-AMI trial: a 43% incidence of the primary composite endpoint is expected in the psychotherapy group and 78% in the control group; therefore, 55 pts will be enrolled to achieve statistical power at the 95% confidence level with 80% power. Given the exploratory nature of the study in the other groups, which renders impossible any hypothesis on the effects of psychotherapy, statistical sample sizing has not been performed in these groups. To match the AMI group, 55 women with BC will also be enrolled. However, only 30 patients with TTS will be enrolled for feasibility reasons, due to the lower percentage of patients admitted to the hospital as compared to the other study groups which could unreasonably slow the enrollment phase. Consequently, it will be necessary to enroll 140 HS in the control group.

### 2.3. Therapies

Drug therapy in both the acute and chronic phase of the illness is left to the treating physician’s discretion and recorded in the case report. Psychopharmacological treatment is not part of this protocol but is recorded in the case report form.

Ontopsychological psychotherapy is a humanistic-existential treatment associated with psychodynamic analysis [29]. It is an original and complex synthesis derived from Freudian psychoanalysis, Jungian analytical psychology, and humanistic-existential psychology, as described by Abraham Maslow [40]. Ontopsychological theory considers the human being as a complex system whereby anything that happens in the body may influence the psyche and vice versa. The bidirectional connections between psyche and body may be considered scientifically based on several studies in the field of psycho-neuro-endocrine-immunology [41,42,43]. In this view, a psychotherapeutic intervention must improve global health to be considered efficacious. This approach includes a thorough analysis of unconscious dynamics, mainly represented by body emotions and dream material [29]. Ontopsychology introduces a new vision of the unconscious dimension, that is sustained by a positive nucleus, which has been called *“In-Self”* (In Sé). It characterizes our specific identity, acting through the vital drive that guides human life, and may be considered the criterion of nature, our specific identity which sustains us throughout life. Other fundamental and distinctive concepts of ontopsychology include: a different and more complex characterization of the *SuperEgo* than in psychoanalysis and a great importance given to the communication process, in particular non-verbal communication, represented both by Physiognomy-Kinesics-Proxemics, and the *semantic field* [44]. The semantic field is a concept developed by pioneers of the ontopsychological school. It is a specific aspect of unconscious communication, a complex interplay of psychic and biological data that emanate unconsciously from every person during all interpersonal interactions.

On the basis of its peculiar topographic and dynamic conception of the unconscious, the meaning of symbolic dream images has also been re-codified according to the theory of interpretation [45], with substantial differences compared to Freudian and Jungian psychoanalytic interpretation.

In particular, the unconscious dimension, sustained by the positive nucleus, In Sé, continuously communicates with the Ego of the person through some specific channels, represented by bodily sensations and emotions, and dreams. The In-Self may be considered a life-long psychic project, whose aim is represented by the optimal psycho-physical evolution of the subject, starting from the biological aspect that is the subject’s health, and extended to all of the personal and social aspects of life.

All patients will undergo counseling for cardiac risk factor modification and a healthy lifestyle. AMI patients will be referred to a CR program.

## 3. Endpoints and Outcome Measures

(A) Qualitative psychological endpoints:

The baseline and follow-up at one and five years of the psychological features in the four groups of subjects (TTS patients, AMI patients, BC women, and HS) as well as subjects classified according to psychotherapeutic treatment will be assessed by the per-protocol psychometric tests, the two enrollment questionnaires (see below), and the Six Drawing test (6DT).

The following qualitative psychological variables will be evaluated at enrollment and the one-year follow-up:

- Perception of body and emotions; qualitative analysis will be performed with two questionnaires, administered to all participants: (1) Enrollment body perception questionnaire (Supplementary Material S1); (2) One-year follow-up body perception questionnaire (Supplementary Material S2).
- Subject’s memory of last week, last month, last year, and past years’ dreams only at enrollment. A qualitative analysis will be performed with two questionnaires, administered to all participants: (1) Enrollment dream questionnaire (Supplementary Material S3); (2) One-year follow-up dream questionnaire (Supplementary Material S4).
- 6DT patterns characteristics

(B) Medical endpoints

Based on the patients’ choice after enrollment, if the size of groups undergoing psychotherapy and those declining is sufficient to allow comparisons, the following medical endpoints will be assessed:

- In AMI and TTS pts: the cumulative incidence of new cardiac events (i.e., MI, revascularization, life-threatening ventricular arrhythmias, recurrence of typical angina pectoris, stroke, and death all-cause), plus the occurrence of any clinically significant new medical disorder (active diseases requiring hospitalization or chronic medical therapy, or that cause a transient or permanent physical limitation), at the one and five-year follow-up in patients undergoing psychotherapy plus standard medical therapy and those on standard medical therapy only.
- In BC patients: the incidence of breast cancer relapse, metastasis, or the occurrence of any clinically significant new medical disorder (that is an active disease requiring hospitalization or chronic medical therapy, or that cause a transient or permanent physical limitation), at the one and five-year follow-up in patients undergoing psychotherapy plus standard therapy and those on standard therapy only.
- In all pts: new hospital admissions.

### 3.1. Tests

The following psychometric tests, previously used in the STEP IN AMI trial, will be administered to all subjects in all four groups at enrollment and at the one and five-year follow-up: the Self-evaluation test, assessing the global level of psychological distress over the preceding two weeks [46]; the Social Support Questionnaire, evaluating the individual’s perception of his/her social network [47,48]; and the Beck Depression Inventory (BDI), evaluating symptoms of major or minor depression [30,31], where a score between 10 and 15 is considered indicative of mild depression symptoms, and a score equal or higher than 16 indicates clinically significant depression. In addition, the State-Trait Anxiety Inventory (STAI) [49] and State-Trait Anger Expression Inventory (STAXI 2) [50] will also be administered at the same time points.

Quality of life will be evaluated at enrollment and after one and five years in AMI, TTS, BC patients, and HS. In patients with AMI and TTS, the Mac New Heart Disease Health-Related Quality of Life Questionnaire will be utilized to assess three specific domains: emotional, physical, and social, as well as a global QOL score [33].

In women with BC two specific psychometric tests evaluating quality of life have been selected: the EORTC QLQ-C30 (European Organization for Research and Treatment of Cancer Quality of Life- Cancer 30, version 3) [51] and the QLQ-BR23 (Quality of Life Questionnaire for Breast cancer 23) as a supplement for assessing specific quality of life issues relevant to patients with breast cancer [52]. The QLQ-C30 incorporates nine multi-item scales: five functional scales (physical, role, cognitive, emotional, and social); three symptom scales (fatigue, pain, nausea, and vomiting); and a global health and quality of life scale. The QLQ-BR23 consists of 23 items covering symptoms and side effects related to different treatment modalities, body image, sexuality, and future perspective.

In HS the Short Form 36 (SF 36) Health Survey Questionnaire will be administered at enrollment and at the one and five-year follow-up [53,54]. It is a 36 item questionnaire, which measures eight variables: physical functioning, social functioning, role limitation due to physical problems, role limitations due to emotional problems, mental health, energy and vitality, bodily pain, and general perception of health. A single item assesses changes in the respondent’s health over the past year. Final scores range from 0 (worst possible health) to 100 (best possible health). Two summary scores are produced: the Mental Health Component Score (which is the result of mental health, role limitations due to emotional problems, and social functioning scores) and the Physical Component Score (which is the result of physical functioning, role limitation due to physical problems, and pain scores) [55].

At enrollment, two qualitative questionnaires are timetabled for all four groups: (1) the perception of body and emotions (enrollment body perception questionnaire; Supplementary Material S1) (2) the subject’s memory of last week, last month, last year, and past years’ dreams (enrollment dream questionnaire, Supplementary Material S3).

At the one-year follow-up, the 1-year follow-up body perception questionnaire (Supplementary Material S2); and the 1-year follow-up dream questionnaire (Supplementary Material S4) are scheduled for all four groups.

For patients, who have undergone psychotherapy, the oneiric material collected during the training will be reported in the section of last years’ dreams. In particular, these two questionnaires will permit evaluation of qualitative changes during psychotherapy, compared to the control group. These two qualitative questionnaires have been specifically developed for this research. The open questions listed in both have been elaborated based on the observations that emerged from the psychotherapeutic practice in the STEP IN AMI trial. For this reason, they are original and their validity will be tested for the first time.

The enrollment dream questionnaire (Supplementary Material S3) retrospectively explores dream patterns from childhood and adolescence, as specified above.

The 6DT will be administered to all subjects at enrollment and the one-year follow-up.

The 6DT is a projective non-structured test defined by the ontopsychological school allowing for a global view of the subject’s psychological and existential situation. It was used to guide therapy in the STEP-IN-AMI trial [27,28]. In this test, the subject is given six blank sheets of paper on which he/she is asked to draw six pictures in the following order: a tree, a person of the same sex, a person of the other sex, a family, the present situation and the future situation. A detailed description of the test can be found elsewhere [27,28,33].

The 6DT is scheduled to be administered to all subjects at enrollment and the one-year follow-up.

### 3.2. Study Design

**Figure 1:**
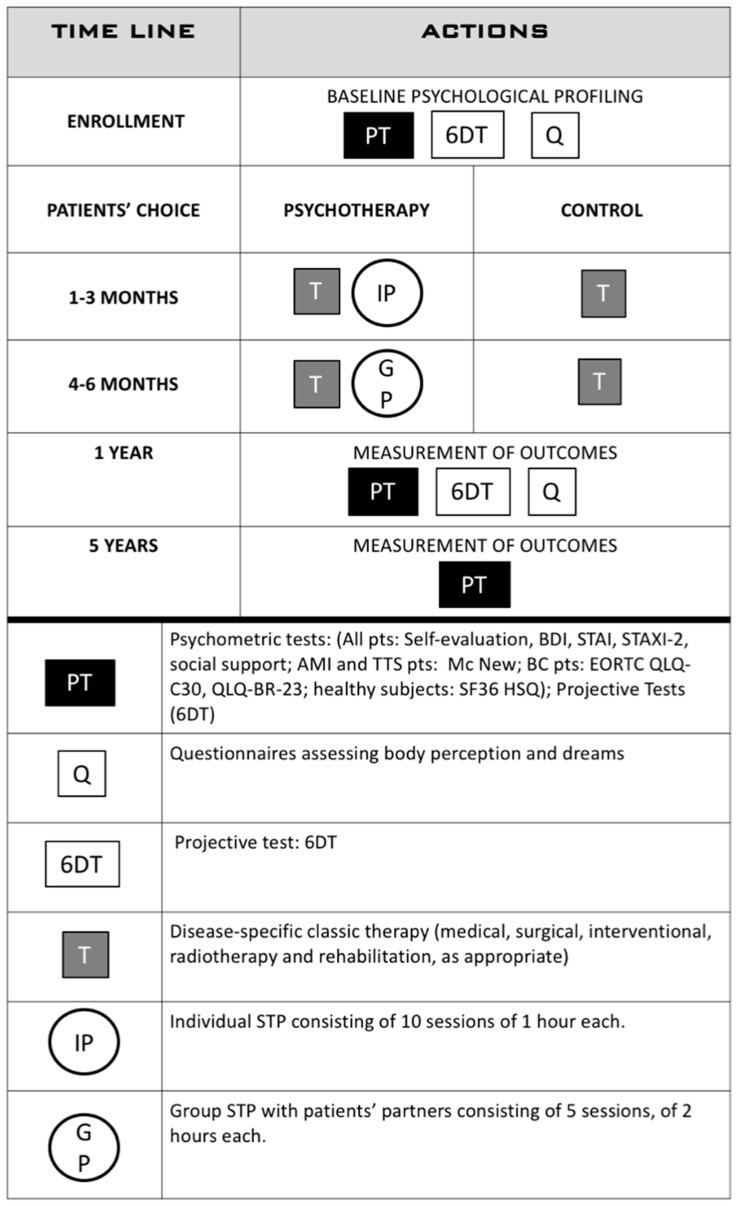
The general layout of the study.

The research is organized into the following phases (Figure 1):

#### 3.2.1. Enrollment

The first phase requires two or three meetings. In the first meeting, the following data will be collected:

(a) Personal demographics and clinical history;
(b) Personal history: patient’s place and date of birth; details about both parents, whether they are alive or their age of death, cause of death, previous and/or current job, their number of siblings (including birth order), patient’s education; previous and current occupation; marital status and age when married; the age of their partner and his/her name, education, and occupation; their number of children, their names, ages, education level, and occupation; favorite personal hobbies; and any other relationships or information about their personal life that the patient feels is relevant to their health and well being.
(c) Psychometric tests (see above);
(d) The Six Drawing test (6DT).

All data will be kept in a dedicated folder and subsequently transferred to an electronic database.

In the following two encounters, the two qualitative questionnaires will be administered to all participants. Differences and peculiar characteristics, in particular, will be evaluated for in the psychometric tests, 6DT, and the two enrollment questionnaires.

#### 3.2.2. Treatment Choice, Implementation, and Group Allocation

In the second phase, all pts with TTS, AMI, and BC will be asked to freely choose between ontopsychological STP along with standard medical therapy and, whenever indicated, rehabilitation therapy or medical therapy plus rehabilitation alone.

Therefore, this phase results in seven study groups: three groups of pts (AMI, TTS, and BC) undergoing psychotherapy, three groups of pts (AMI, TTS, and BC) on standard medical therapy, and one group of HS.

There will be four independent areas of research: (1) AMI pts following psychotherapy (AMI-PSY) will be compared with AMI pts, without psychotherapy (AMI-C); (2) TTS pts who will follow psychotherapy (TTS-PSY) will be compared with TTS pts without psychotherapy (TTS-C); (3) BC pts with psychotherapy (BC-PSY), will be compared with BC pts without psychotherapy (BC-C); (4) HS subjects, after the initial comparative evaluation with the other enrolled groups, will have an independent follow-up, where psychological data collected at enrollment will be compared with psychological data collected at one year and with clinical follow-up at five years.

Since the research is observational, it is not possible to foretell how many patients will choose psychotherapy plus medical therapy or standard medical care alone.

If balanced patient groups can be formed, statistical analysis evaluating differences between groups will be performed. Thus, it may be possible to evaluate how psychotherapy may impact the real world because patients will have a choice between psychotherapy plus medical care or medical care alone. It will also be possible to evaluate if psychotherapy might result in an improvement in psychological patterns compared to medical therapy alone, and how this can translate in an improvement in medical prognosis. Moreover, the patients’ choice might provide useful information to improve a more individualized therapy and hospital organization.

AMI-PSY and TTS-PSY patients will be referred to the outpatient clinic of Cardiac Psychology at San Filippo Neri Hospital in Rome; BC-PSY will be referred to the outpatient clinic of Psycho Oncology in the same hospital. Psychotherapy will be provided in individual sessions (maximum of ten) and five group meetings, as in the STEP IN AMI trial. Psychotherapy will be conducted by two expert psychotherapists trained in ontopsychology and psychosomatics.

HS will not be offered psychotherapy but will be free to obtain private psychotherapy.

#### 3.2.3. Follow-Up

Follow-up is planned at one and five years with a comprehensive medical visit and yearly routine blood tests.

For AMI pts, echocardiography, an exercise stress test, or stress myocardial perfusion scintigraphy, will be performed when medically indicated.

For TTS pts echocardiography is timetabled at one month and six months after the acute phase, and yearly through the five-year follow-up when indicated.

For BC pts, history, physical examination, and mammography will be obtained at follow-up. Physical examination is planned every 3 to 6 months for the initial 3 years, every 6 to 12 months for years 4 and 5, and annually thereafter. For women who have undergone breast-conserving surgery, a post-treatment mammogram is scheduled 1 year after the initial mammogram and at least 6 months after completion of radiation therapy. Thereafter, unless otherwise indicated, a yearly mammogram will be scheduled. The use of complete blood counts, chemistry panels, bone scans, chest radiographs, liver and pelvic ultrasound, computed tomography scans, fluorodeoxyglucose–positron emission tomography scans, magnetic resonance imaging, and/or tumor markers (carcinoembryonic antigen, CA 15-3, and CA 27.29) are not recommended for routine follow-up in an otherwise asymptomatic patient with no specific findings on clinical examination [56].

At the one-year follow-up all patients and HS will be administered: (a) the same psychometric tests administered at enrollment; (b) two qualitative questionnaires, the 1-year follow-up body perception questionnaire (Supplementary Material S2), and the 1-year follow-up dream questionnaire (Supplementary Material S4); (c) the 6DT.

At the five-year follow-up, all psychometric tests will be administered to all of the groups.

Data will be recorded on paper case-report forms and transferred into an electronic database, with the principal investigator cross-checking consistency of data for all patients.

## 4. Statistical Analysis

An external committee will evaluate the results in each phase of the research and the characteristics of each group will be analyzed. The qualitative results related to the 6DT, body perception questionnaire, and dream questionnaire at enrollment and the one-year follow-up. It is impossible to foretell the specific features of oneiric symbols and patterns before the study, as the research is a pilot study in the field of psychosomatics. The oneiric manual of the ontopsychological school [45] will be used as an initial reference point for the analysis and classification of dreams.

Unless otherwise specified, all study data will be analyzed on an intention-to-treat basis.

Event rates will be expressed as proportional incidences and as person−time incidence rates. Continuous variables for each of the study groups will be reported as means (with standard deviations or 95% confidence intervals) or as medians (lower quartile-upper quartile), as appropriate; categorical variables will be reported as absolute numbers and percentages. Continuous unpaired variables will be compared using the independent-sample Student’s t or Mann-Whitney U tests, and continuous paired variables will be compared using paired Student’s t or Wilcoxon tests, as appropriate. Categorical variables will be compared by Pearson’s chi-squared analysis or Fisher’s exact test, as indicated. The outcome incidence within each study group over long-term follow-up will be compared with Friedman’s test. Survival analysis will be performed using Cox proportional hazards ratios. Multivariable binary logistic regression analysis will be performed to appraise the independent predictive role of baseline characteristics on outcomes. Spearman’s rank correlation test will be performed to assess the correlation between variables. A *p* value < 0.05 will be considered statistically significant, with all inferential tests 2-tailed. Statistical analysis will be performed using the statistical software program SPSS, version 11.5 (SPSS Inc., Chicago, IL, USA).

### Enrollment Status

At this time, 75 out of a total of 280 subjects have been enrolled: 19 with TTS (15 have chosen psychotherapy); 11 women with BC (5 have chosen psychotherapy); 4 with AMI (1 has chosen psychotherapy); 41 HS.

## 5. Discussion

The PSYCHONIC study is an observational research protocol, primarily designed to obtain at enrollment a qualitative analysis of distinctive psychological characteristics of patients suffering from different NCDs: ischemic heart disease, Takotsubo syndrome, and breast cancer, and comparing them to age and sex-matched control subjects without major diseases. Some psychological areas are explored systematically for the first time: “body perception”, dream patterns, and the 6DT. We are exploring new hypotheses which may highlight possible specificities or similarities in different clinical presentations.

From a psychoanalytic perspective, and especially for ontopsychology, dream material and analysis are a fundamental part of the theory and for the psychotherapeutic approach. In this observational trial, we hypothesize that comparing psychosomatic characteristics in different clinical settings may shed light on pathophysiologic and clinical issues and provide novel working hypotheses. This study, addressing cardiologic and non-cardiologic syndromes together, may appear non-coherent from a classic perspective. However, this study design has the potential to address possible underlying common pathogenic processes shared among different diseases beyond the reductionist splitting of the human being according to an organ or clinical presentation.

Despite observational studies’ inherent, well-known limitations, they complement randomized controlled trials (RCTs) in several aspects. RCTs are poor in assessing the generalizability of an intervention whereas observational trials can address this issue with an intervention that has been proven internally effective in RCTs. In this case, the STEP-IN-AMI trial provided good randomized evidence of the efficacy of ontopsychological psychotherapy after AMI in selected patients, but it is still unknown to what extent this can be extrapolated to the real world.

The results of the PSYHCONIC study have been designed to shed light on some possible underlying mechanisms and therefore generate new working hypotheses to better target interventions. Therefore, it may help in assessing the efficacy of ontopsychological psychotherapy after AMI in the real world and in preliminarily testing the hypothesis of its efficacy in other NCDs.

## Data Availability

The data presented in this study are available on request from the corresponding author. The data are not publicly available due to privacy and ethical reasons.

## Supplementary Materials

The following are available online at https://www.mdpi.com/article/10.3390/jcm10245786/s1, Supplementary Material S1: Enrollment body perception questionnaire; Supplementary Material S2: One year follow-up body perception questionnaire; Supplementary Material S3: Enrollment dream questionnaire; Supplementary Material S4: 1-year follow-up dream questionnaire.
