# Supplementary material for: PSYChosomatic Medicine in ONcologIc and Cardiac Disease (PSYCHONIC) Study—A Retrospective and Prospective Observational Research Protocol
Source: J Clin Med. 2021 Dec 10;10(24):5786. doi: 10.3390/jcm10245786 (PMC8709231; doi:10.3390/jcm10245786)
Supplement: Supplementary file 1 [file jcm-10-05786-s001.zip › Suppl. Material 1 Enrollment body perception questionnaire.pdf]

**ASL RM1**:
San Filippo Neri Hospital

# PSYCHONIC (PSYChosomatic medicine in ONcologIc and Cardiac disease)
# Observational retrospective and prospective research protocol in psychosomatics: cardiologic and oncologic fields

## ENROLLMENT BODY PERCEPTION QUESTIONNAIRE

**Name** **Surname**

Dear Mrs/ Mr

As already explained by the doctor and by the psychologist during the first meeting of the enrollment phase of the PSYCHONIC research, one of the aims of this study is to evaluate body and emotions perception in patients with cardiac disease, in patients with oncologic diseases and in a control group of subjects without clinical evidence of disease.

For this reason we ask you to listen to your body sensations and emotions during a few minutes of meditation. After we well ask you to answer to four simple questions.

1) Which is your state of mind in this moment?

2) What parts of your body can you be aware of?

3) What parts of your body do you feel as extraneous or you cannot be aware of?

4) Regarding the parts of your body that you are aware of, what sensations do you feel? Pain, comfort, etc.

Now you are invited to do a brief exercise of body relaxation for ten minutes. You are invited to choose a comfortable body position, to close your eyes and breathe calmly under your psychotherapist guidance.

At the end of this exercise we well ask you to answer to the same four simple questions of the first part of the questionnaire.

5) Which is your state of mind in this moment?

6) What parts of your body can you be aware of?

7) What parts of your body do you feel as extraneous or you cannot be aware of?

8) Regarding the parts of your body that you are aware of, what sensations do you feel? Pain, comfort, etc.

We thank you very much for your kind collaboration
