# Supplementary material for: PSYChosomatic Medicine in ONcologIc and Cardiac Disease (PSYCHONIC) Study—A Retrospective and Prospective Observational Research Protocol
Source: J Clin Med. 2021 Dec 10;10(24):5786. doi: 10.3390/jcm10245786 (PMC8709231; doi:10.3390/jcm10245786)
Supplement: Supplementary file 1 [file jcm-10-05786-s001.zip › Suppl. Material 4- 1-year follow-up dream questionnaire.pdf]

ASL RM1:
San Filippo Neri Hospital

PSYCHONIC (PSYChosomatic medicine in ONcologIc and Cardiac disease)
Observational retrospective and prospective research protocol in psychosomatics: cardiologic and oncologic fields

1-YEAR FOLLOW-UP DREAM QUESTIONNAIRE:

Name **Surname**

Dear Mrs/ Mr

As already explained by the doctor and by the psychologist during the enrollment phase of the PSYCHONIC research, one of the aims of this study is to evaluate the dreams pattern in patients with cardiac disease, in patients with oncologic diseases and in a control group of subjects without clinical evidence of disease; to clarify the characteristics of oneiric symbols related to specific pathologies under examination, compared to those of a "healthy subjects" group. In this way it is possible to verify if oneiric symbols differ in the different areas under examination and if they might have a predictive role related to the onset of the disease.
This knowledge might contribute to a better comprehension of psychosomatic process and to improve the medical and psychotherapeutic approach in these patients.

For this reason we ask you to tell the dreams, that you remember related to different periods of your life.

1) **DREAMS OF LAST WEEK**

2) **DREAMS OF LAST MONTH**

3) **DREAMS OF LAST YEAR**
